# Kohonen Network-Based Adaptation of Non Sequential Data for Use in Convolutional Neural Networks

**DOI:** 10.3390/s21217221

**Published:** 2021-10-29

**Authors:** Michał Bereta

**Affiliations:** Department of Computer Science, Cracow University of Technology, ul. Warszawska 24, 31-155 Kraków, Poland; michal.bereta@pk.edu.pl

**Keywords:** kohonen network, convolutional neural network, multiple input neural networks

## Abstract

Convolutional neural networks have become one of the most powerful computing tools of artificial intelligence in recent years. They are especially suitable for the analysis of images and other data that have an inherent sequence structure, such as time series data. In the case of data in the form of vectors of features, the order of which does not matter, the use of convolutional neural networks is not justified. This paper presents a new method of representing non-sequential data as images that can be analyzed by a convolutional network. The well-known Kohonen network was used for this purpose. After training on non-sequential data, each example is represented by so-called U-image that can be used as input to a convolutional layer. A hybrid approach was also presented, where the neural network uses two types of input signals, both U-image representation and the original features. The results of the proposed method on traditional machine learning databases as well as on a difficult classification problem originating from the analysis of measurement data from experiments in particle physics are presented.

## 1. Introduction

Convolutional neural networks (CNN) have been used successfully to tackle the complex problems of classifying and processing images, time series, and other sequential data. They are one of the sources of deep learning success. Sequential data are often the result of measurements with a variety of sensory devices such as cameras or ECGs. However, in many situations, the measurement result is described by a feature vector, which is not sequential. Its components are not related by spatial correlation of their location in the vector. There is no justification for using the CNN network for such data. In this work, however, we propose a technique for adapting non-sequential data to such a form that they can be used as input signals to the convolution layer. For this purpose, the Kohonen network is trained first based on original data in the form of feature vectors. Then, each feature vector is described with a special image, called a U-image, which is generated using a trained Kohonen network. Such an image can be interpreted as an alternative representation of an object originally described by a feature vector. It can be used alone as an input to the convolution layer. It can also be utilized in conjunction with the original feature vector to enrich its representation. In this case, it is possible to use a multiple-input neural network, which accepts as input both the original features and the representation with the generated U-image. Both of these approaches are described in detail and tested in this paper. The performed numerical experiments show that in many cases, the improvement of the classification model’s quality is possible compared to the traditional feed-forward neural network architecture applied to data in the form of a feature vector.

Later in this section, we present briefly related work and list the central innovative ideas proposed in this paper. The second section presents the most important information about Kohonen neural networks and presents the basic learning algorithm. We remind the definition of the U-matrix, as well as introduce the definition of the U-image object. The method of using U-images as input signals to the convolution layer is described in the third section. The fourth section presents numerical experiments that confirm the benefits of using the proposed computational technique. The fifth section concludes the work.

### 1.1. Related Work

In this work, we propose to combine the functionality of convolutional networks [1] and Kohonen networks [2]. Each of these networks has played an essential role in the development of computational intelligence methods. CNN networks are one of the primary sources of deep learning success. They have been used in image recognition [3,4,5], facial analysis [6], speech recognition [7], analysis of ECG records [8,9], analysis of medical images [10], natural language processing [11], and many other problems of classification of sequential data, i.e., videos, images and time series. Their key role in computational generative methods such as autoencoders [12] and generative-adversarial networks (GANs) [13] cannot be overlooked either. The use of convolutional filters allows for a significant saving of memory, which allows the construction of larger models. On the other hand, Kohonen networks played a significant role in unsupervised learning algorithms in problems of data analysis and visualization. One of the methods of data visualization using the Kohonen network, called U-matrix, has been described in [14,15]. It is an inspiration for the U-image proposed in this work. It is impossible to thoroughly review the techniques involved in both network types and their wide application to many problems in this work. However, it is worth emphasizing that they are combined innovatively to enable their even more comprehensive application in this work.

### 1.2. Proposed Innovative Solutions

Both Kohonen networks and convolutional networks have been known for years. An innovative computational technique proposed in this work is their joint use in the case of classification problems of non-sequential data, i.e., data that should not be directly used as input signals to the convolutional layer. For this purpose, a definition of a new U-image object was proposed, which, although defined in a manner similar to the previously known U-matrix, is a new and important idea in the proposed approach. This work also shows how to combine the original data and the U-image representation for building a multi-input network. Based on the available knowledge, it can be stated that the computational technique proposed in this paper is innovative and has no equivalent in the published works.

## 2. Kohonen Networks

Kohonen networks [2], also called Kohonen maps, are neural networks that are used to prepare a low-dimensional (usually two-dimensional) representation of high-dimensional data. They are often used in data visualization. Unlike most of the neural networks used in practice, which are trained using gradient supervised learning, Kohonen networks use unsupervised, competitive learning. Learning aims to preserve the topological similarity of high-dimensional input data and reflect it on the so-called map, which is usually a two-dimensional representation of the original data. Data vectors close to each other in the original multidimensional space are mapped to Kohonen’s map areas, preserving this relationship. Maps usually have a one-dimensional or two-dimensional structure. Typically, a two-dimensional grid of neurons is assumed.

In this work, we consider the Kohonen network in the form of a two-dimensional neural network. Each neuron has two characteristics, a weight vector and its coordinates in a two-dimensional network of neurons. The length of the weight vector is equal to the dimensionality of the original high-dimensional data. The original data space is called the input space, while the two-dimensional grid of neurons is called the map space.

Suppose we have training data in the form of $\left\{x_{i}\right\}$, where $x_{i}\in R^{N}$. The training of Kohonen neural network is based on modifying the weights of neurons and is competitive. Starting with random initial values, neuron weights are updated several times based on the training examples. In each epoch, for each training example, BMU (Best Matching Unit) is found with the weight vector with the minimum Euclidean distance to the feature vector of the training example. BMU weights are modified as well as the weights of its neighbors found on the 2D grid. The definition of the neighborhood is not uniquely given. In the case of a 2D grid, it can be the set of its direct four or eight neighbors. A function $\theta (i,j,i_{BMU},j_{BMU})$ can also be defined, which will assign each neuron (i,j) the degree of its neighborhood, depending on its distance from the BMU on the 2D mesh. A common approach is to decrease the value of the neighborhood function as the distance from the BMU and the degree of advancement of the training process increase.

The basic training procedure for Kohonen map is given as Algorithm 1.

**Algorithm 1:** Training of Kohonen network

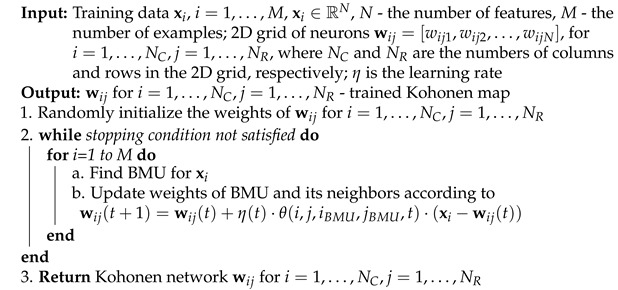



### U-Matrix and U-IMAGE

**Figure 1:**
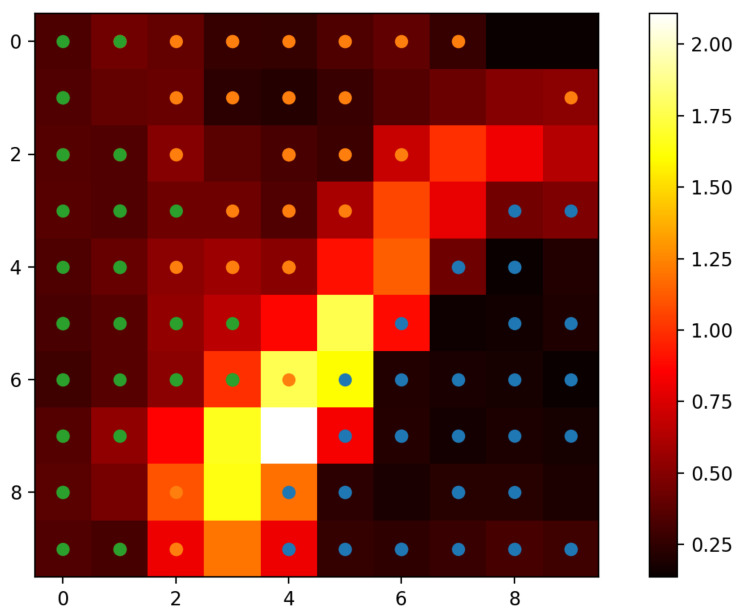
U-map for iris data with the examples mapped as green, orange and blue dots.

A trained Kohonen neural network is typically used for data visualization. An example of such a visualization is presented in Figure 1. Data from the Iris database were used to train the network. The three classes of iris species are represented by three colors. Each example is originally described by four features. Therefore, it is not possible to visualize the data in the original space. However, on the Kohonen map for each iris example, you can mark its position on the 2D grid by finding the neuron that is its BMU. You can see that the objects of each class that are close to each other in the original 4D space are grouped close to each other on a 2D grid, i.e., the learned projection retains topological similarity.

For a trained Kohonen neural network one can define the so-called U-map (U-matrix) [14,15]. Each neuron presented as a pixel in a 2D image can be assigned a color depending on the average distance of its weight vector from the weights of its neighboring neurons in a 2D grid. Such visual information can be used to determine which adjacent neurons on a 2D grid also form groups in a high-dimensional space. In the example U-map shown in Figure 1, dark color means that a given neuron has weights similar to the weights of its neighbors, while light means that despite its proximity in a 2D grid, it is distant from them in the high-dimensional space. In the example of a network trained on the Iris data, this allows us to observe the fact that one of the classes is very well separated from the other two in the original 4D space.

**Figure 2:**
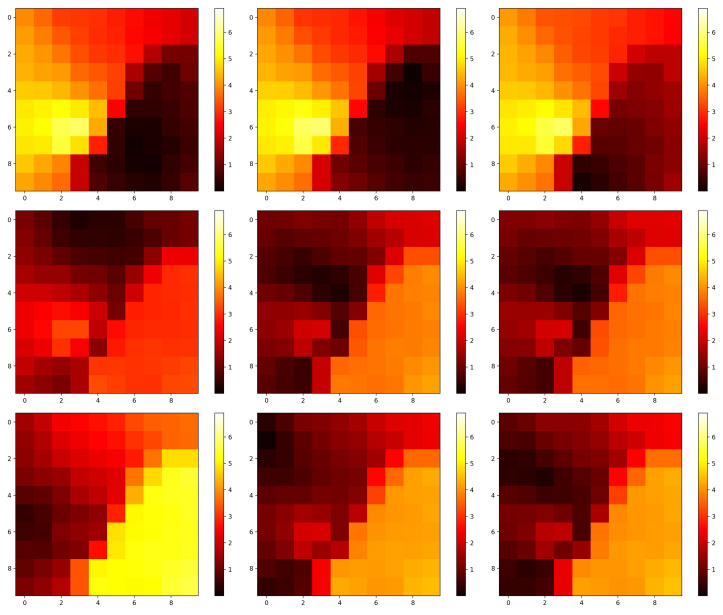
U-images for example iris data. Each row contains three examples from a given class.

One of the new ideas proposed in this paper is to define a new object called U-image. The U-map is defined for the trained Kohonen neural network. We define the U-image for each example, training or testing, on the basis of a trained Kohonen map. At this point, we propose, by analogy with the U-map creation process, to define the U-image as follows. For a given $x_{i}$, training or test example, prepare a U-image by assigning a value to each neuron in a 2D network equal to the Euclidean distance of that neuron’s weights from the feature vector $x_{i}$. These values, properly scaled, can be used to present $x_{i}$ as a 2D image, without no matter what the original dimensionality of $x_{i}$ is. The procedure for creating a U-image is presented as Algorithm 2. Figure 2 shows in each row three examples from a different class in the iris database, presented as U-images. Clearly, visualization with U-images is able to capture the similarity of examples from the same class as well as differences between classes.

**Algorithm 2:** Calculation of U-image

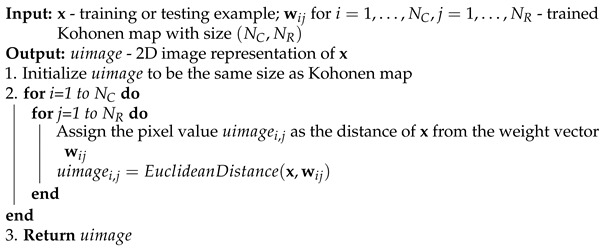



## 3. Proposed Method

**Figure 3:**
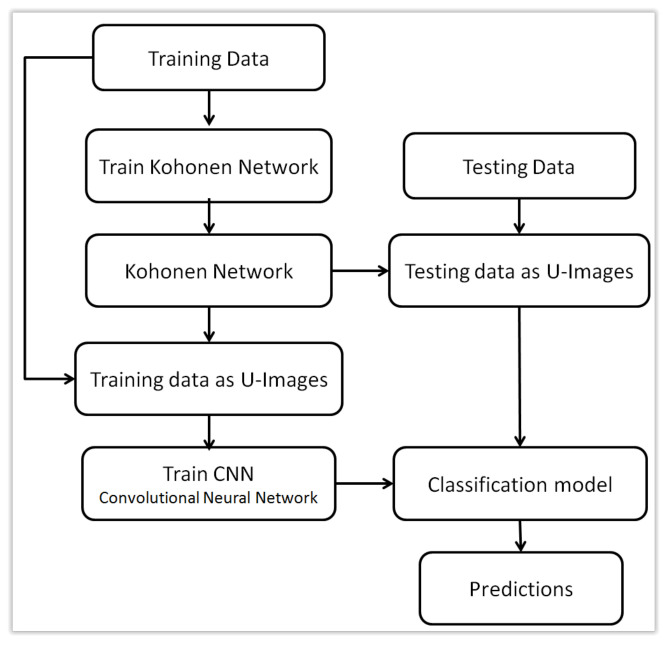
Using U-images to train Convolutional Neural Network.

**Figure 4:**
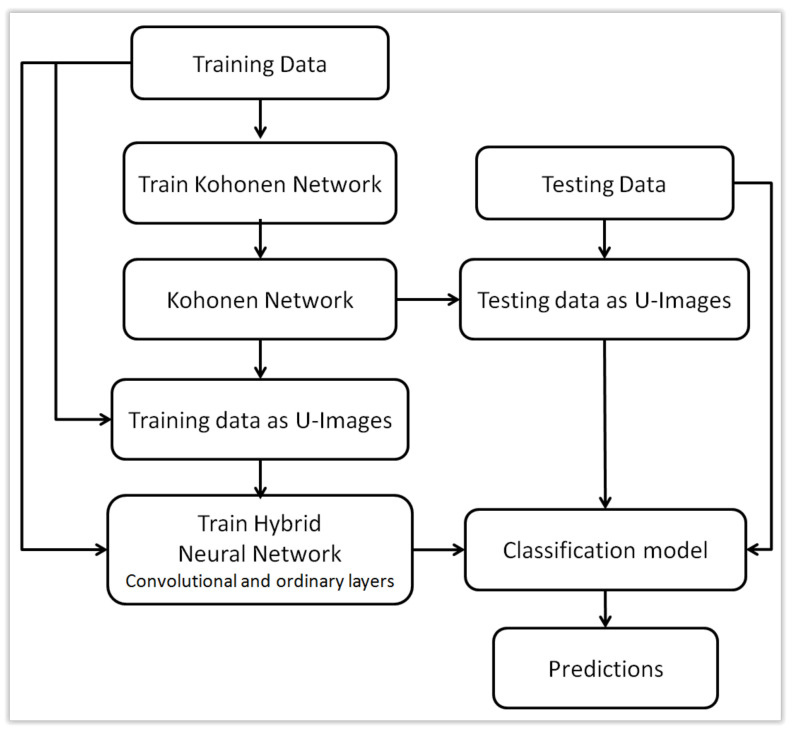
Using both original data and U-images to train hybrid (multi-input) neural network with convolutional layers.

**Figure 5:**
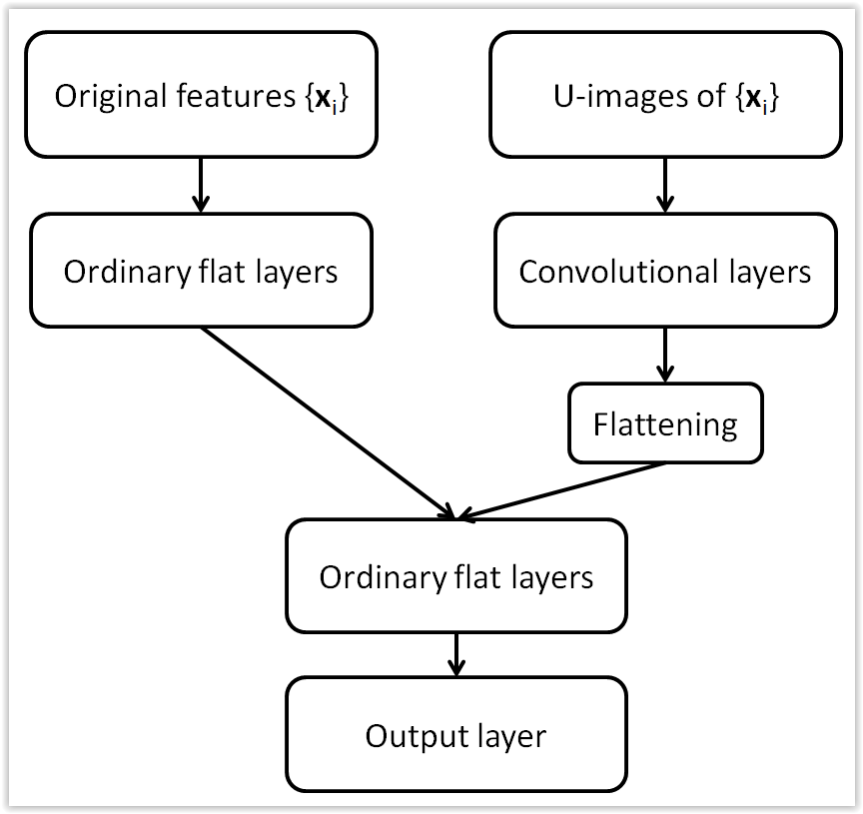
Architecture of a hybrid (multi-input) neural network with two types of input data.

U-images obtained for examples $x_{i}$ based on a previously trained Kohonen map can be treated as a new, alternative form of representation of the original data $x_{i}$. These are images, so although the original data was not sequential, now the new representation allows them to be used as input to convolutional layers in a neural network. This idea is the main novelty presented in this work. This approach will be presented in two versions. First, U-images can replace the original data $x_{i}$, which makes it possible to use a traditional convolutional layer whose input are images. This approach is schematically presented in Figure 3. The second approach, which can be called hybrid, combines the original $x_{i}$ representation with a new representation using images. This idea is presented in Figure 4 while the architecture of a hybrid neural network with two types of input data is presented in Figure 5. Both forms of representation are used as input to the neural network, with images being processed by convolutional layers and the original $x_{i}$ representation by ordinary flat layers of neurons.

The proposed method has two components, the use of a Kohonen network and the use of a convolutional neural network. The computational complexity is greater compared to using only the neural network with the original attributes. This is due to two factors. First, the need to train the Kohonen network and use it to compute the U-image representation. Second, a convolutional network will usually have more parameters than a traditional feed-forward network. In the case of Kohonen network training, the phase of calculating the distances between training data and weight vectors in one iteration of the main training loop has the complexity $O(M\cdot N\cdot N_{C}\cdot N_{R})$, where *M* is the number of training examples, *N* is the number of original features, $N_{C}$ and $N_{R}$ are the number of columns and rows, respectively, in the Kohonen network. The BMU search step for each training example has a complexity of $O(M\cdot N_{C}\cdot N_{R})$. The step of updating the weights of each neuron for each example has a computational complexity of $O(M\cdot N\cdot N_{C}\cdot N_{R})$. Additionally, computing a U-image representation for each example has a complexity of $O(M\cdot N\cdot N_{C}\cdot N_{R})$. Some steps can be optimized, for example by using the spatial indexing method such as R-Tree, the cost of finding the BMU for a single training example can be reduced to $O(log\left(N_{C}\cdot N_{R}\right))$. Considering that after training the Kohonen network, only the step of calculating the U-image representation is necessary on the test data, it can be implemented on low powered devices and sensors. However, the application of the proposed method on such devices depends on the convolutional architecture of the neural network used. In many cases, it is necessary to apply appropriate compression of such a network, for example by using dynamic quantization techniques on different layers. Examples of such implementations can be found in works [16,17].

## 4. Numerical Experiments

This section presents the results of a comparative analysis of the different neural architectures proposed in this work. Known and frequently used datasets from the UCI Machine Learning Repository were used in the numerical experiments [18]. Sixteen classification problems with two classes were selected. Table 1 summarizes the data showing the number of examples, the number of attributes, and the majority class ratio for each dataset. The datasets represent classification problems of varying degrees of difficulty. Their common feature is that a set of attribute values represents each example, so they are not described with sequential data that could be directly used as input to the convolutional network.

### 4.1. Methodology

We compared the algorithms with 10-fold cross-validation reporting mean test classification errors as the average of the test errors of the different folds. The divisions into ten folds were the same for all algorithms. We compared four different neural designs. First, the traditional feed-forward neural architecture with the original attribute vectors as input. Second, the proposed architecture, which is a convolutional neural network accepting U-images as inputs. In this approach, we test the usefulness of hidden convolutional layers instead of flat hidden layers. The third architecture is the extension of the proposed approach, i.e., we use U-images as inputs to CNN. However, we also use additional flat hidden layers together with convolutional layers. The fourth approach is based on a multiple-input neural network, which accepts both the original input (vector of features) and the U-images produced by the Kohonen network. Thus, both convolutional and traditional flat layers are used in this approach. In each approach, we tested several architectures by specifying the number of hidden layers of a given type and the number of neurons in each layer. For each approach and each dataset, we report the best-performing architecture. The corresponding sections give details about the tested neural networks. In each neural network we used ReLU activation function, softmax classification layer, categorical crossentropy as the loss function and Adam optimization algorithm to tune the weights for 100 epochs.

### 4.2. Ordinary Feed-Forward Neural Networks

**Figure 6:**
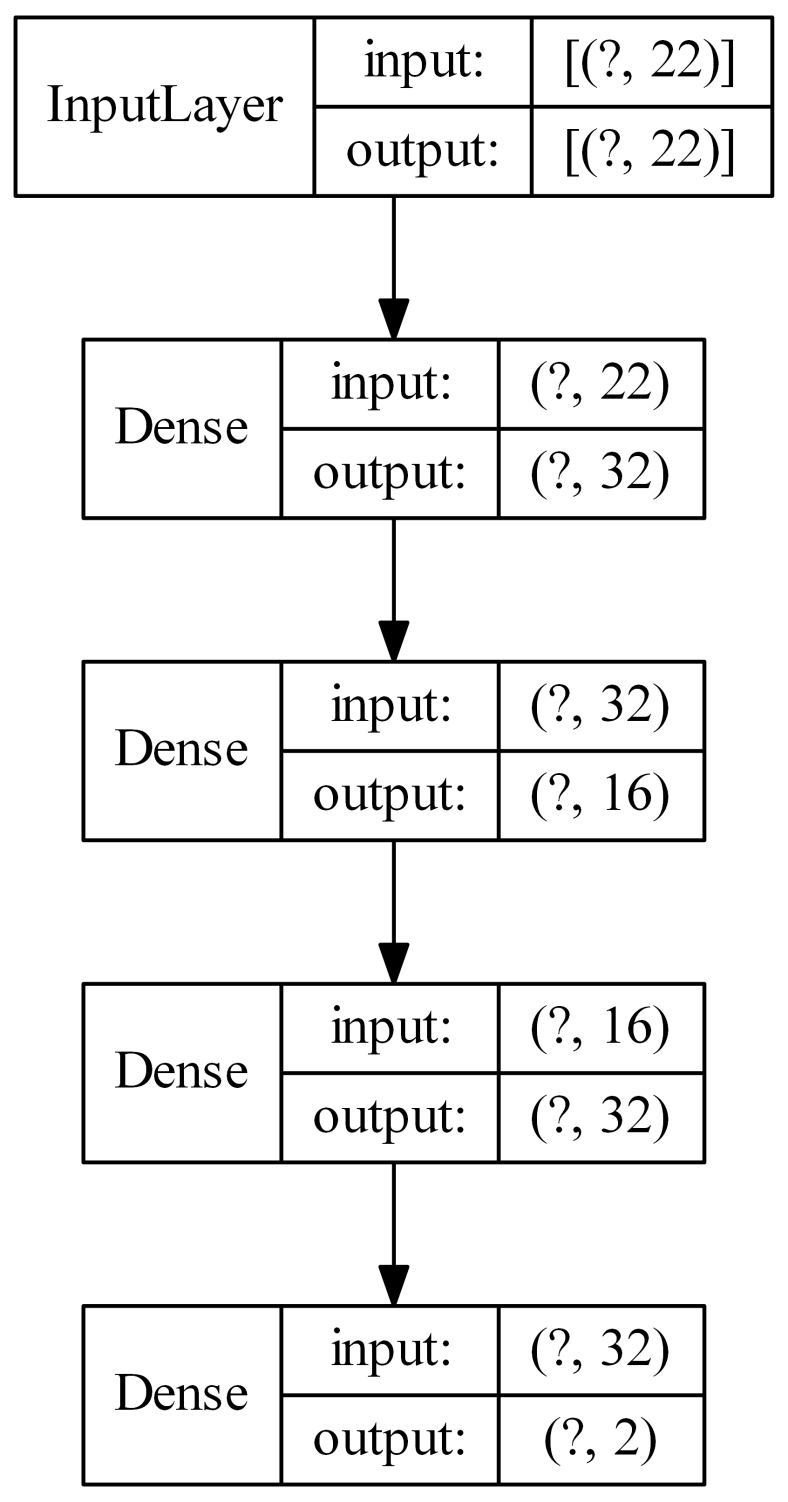
Example architecture of an ordinary feed-forward neural network (the question mark represents the number of examples).

For each dataset, the following neural architectures of ordinary feed-forward neural networks were tested: 32, 16-32, 32-32, 16-16-32, 32-16-32, 32-32-32, where the values represent the numbers of neurons in successive hidden layers. Thus, for example, 32-16-32 describes a neural network with three hidden layers with the number of neurons in each successive hidden layer 32, 16 and 32, respectively. The input to the networks were vectors of features. Figure 6 presents such a model configured for parkinsons dataset (22 original features).

Table 2 presents the mean test errors. For each dataset we report also the architecture with the best results.

### 4.3. Kohonen and Convolutional Neural Network

**Figure 7:**
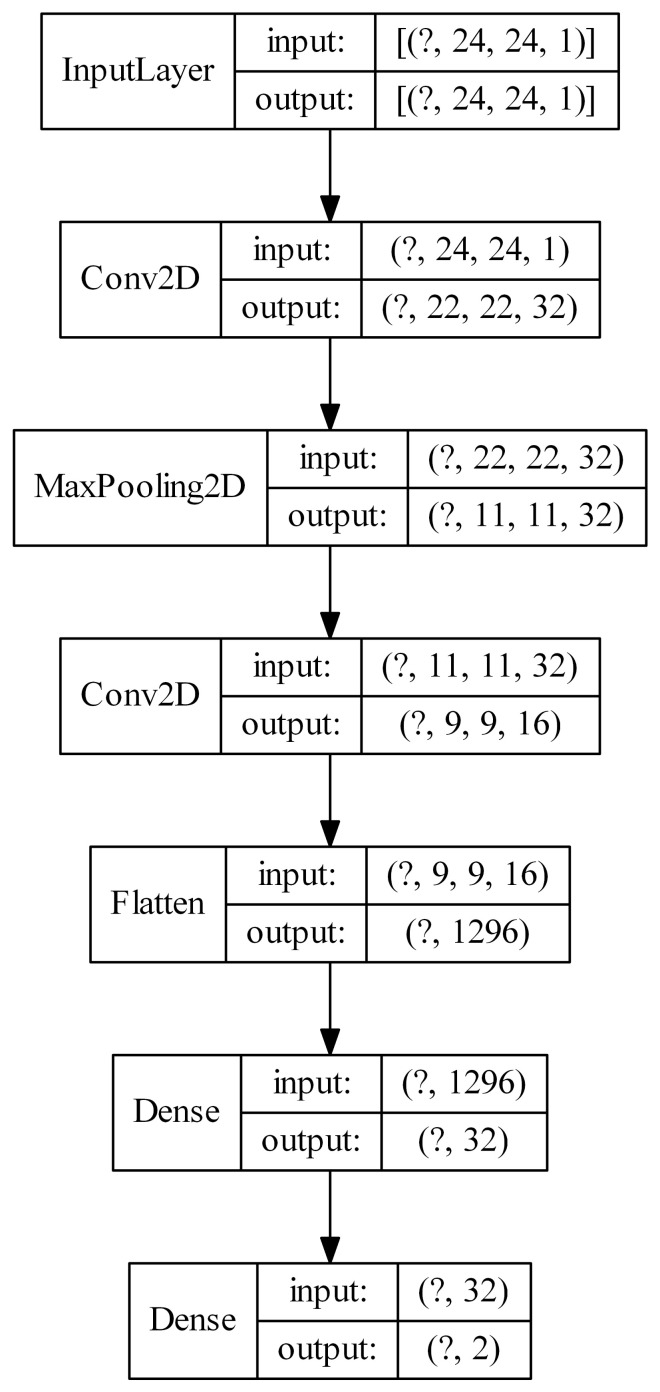
Example architecture of a convolutional neural network with U-images produced by means of a Kohonen network as input (the question mark represents the number of examples).

In this section, we present the results of the core idea of this work. The original feature vectors in each dataset are first used to train Kohonen network. Then, the U-images are generated and used alone to train the convolutional neural networks. The U-images are the only type of input for the classification model (CNN) in this set of experiments. It is worth to mention, that a separate Kohonen network is trained in each iteration of the 10-fold cross-validation. The following architectures of Kohonen-CNN models were tested: k16 × 16-cnn16, k20 × 20-cnn16, k24 × 24-cnn16, k16 × 16-cnn32-16, k20 × 20-cnn32-16, k24 × 24-cnn32-16, k30 × 30-cnn32-16, k30 × 30-cnn32-32. In the description, first the size of the Kohonen network is given (16 × 16, 20 × 20, 24 × 24 or 30 × 30), then the information about the number of convolutional filters of size 3 × 3 in successive hidden layers. Max pooling operation of size 2 × 2 was applied after each, but not the last one, convolutional layer. After the last convolutional layer and before the classification layer, a flattening operation was applied and one ordinary flat layer of 32 ReLU units was used. For example, k24 × 24-cnn32-16 describes a model, in which first a Kohonen network of size 24 × 24 was trained and used to calculate U-images. They were then applied as input to a CNN with two convolutional layers, first with 32 filters (with max pooling), and the second with 16 filters. After those, there is a flattening, an additional flat layer of 32 ReLU units, and the classification layer. Figure 7 presents such a model. Note that Kohonen network itself is not presented.

**Figure 8:**
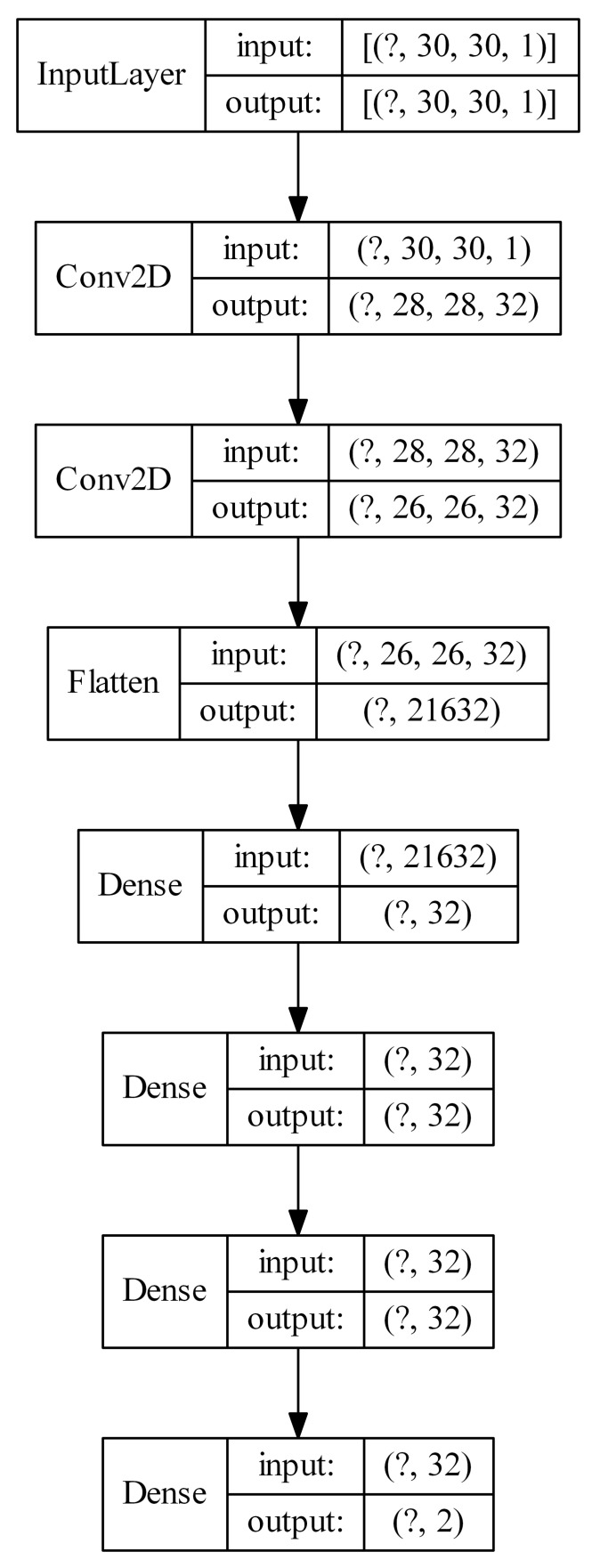
Example architecture of a convolutional neural network with U-images as input, together with additional hidden flat layers (the question mark represents the number of examples).

**Figure 9:**
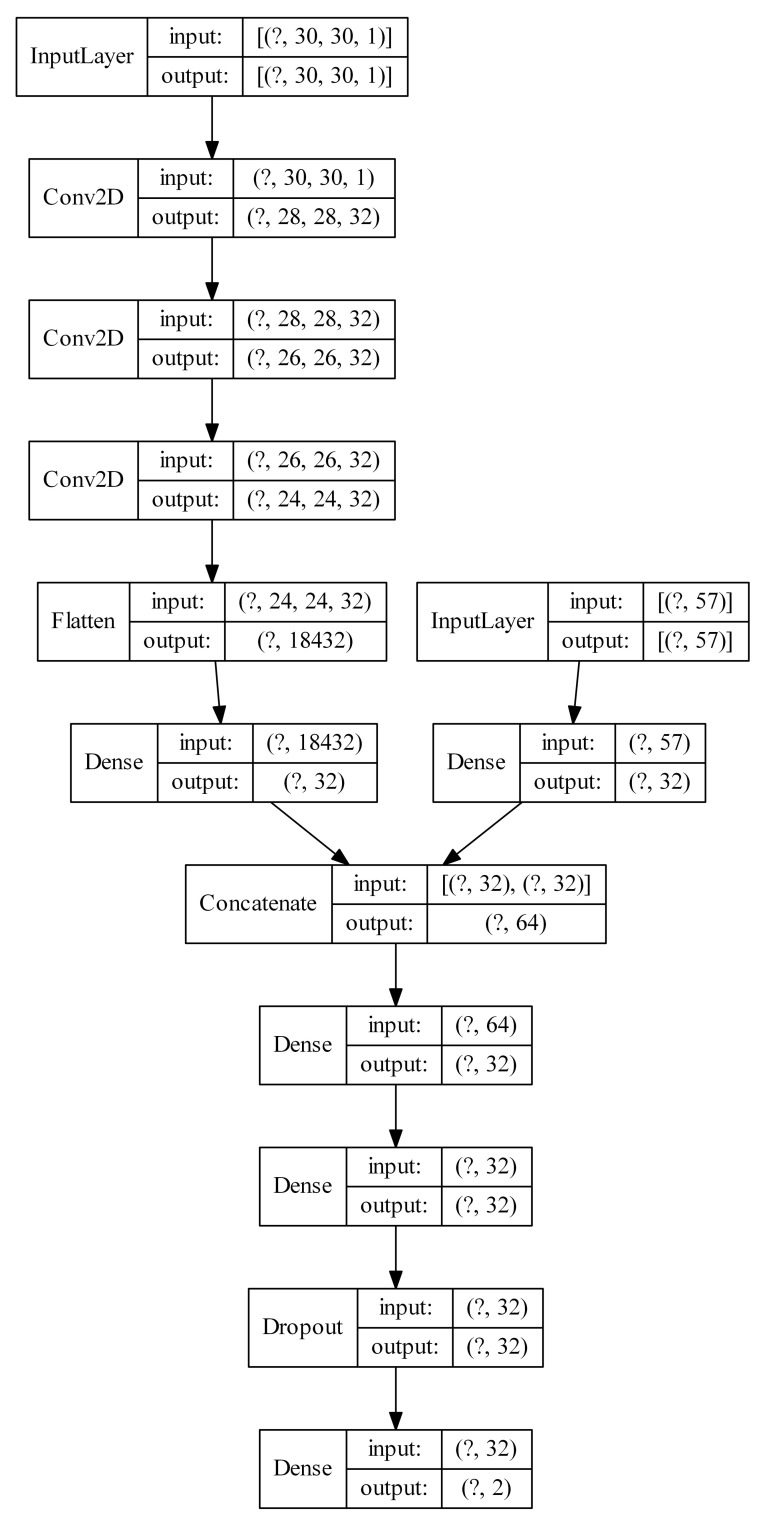
Example architecture of a multiple input neural network (the question mark represents the number of examples).

In the neural network diagrams shown in Figure 6, Figure 7, Figure 8 and Figure 9, the sizes of the tensors describing the input and output of a given layer are shown in parentheses. The question mark represents the number of training/test examples. In general, it is not known, so the question mark acts as a placeholder. For example, in Figure 7, the input of the first convolutional layer is described by a tensor of size (?, 24, 24, 1), which means that the network input is an image with a size of 24 × 24 pixels and one channel (in this case it is a U-image obtained from Kohonen’s map with a size of 24 × 24 neurons). Thirty-two feature maps, each of the size of 22 × 22 pixels, are the output of this layer.

Table 3 presents results for Kohonen-CNN networks. The best performing architectures are reported.

### 4.4. Kohonen and Convolutional Neural Network with Additional Flat Hidden Layers

In this section, we test models very similar to those described in the previous section. The difference is that we experimented with the additional flat hidden layers after the convolutional layers. For each Kohonen network size (k16 × 16, k20 × 20, k24 × 24 and k30 × 30), we tested the following CNN architectures: cnn16-nn32, cnn16-nn32-32, cnn32-nn32, cnn32-nn32-32, cnn32-32-nn32, cnn32-32-nn32-32. They should be understood as first giving the number of filters in the successive convolutional layers (e.g., cnn32-32), then the number of ReLU units in the flat hidden layers after the flattening operation (e.g., nn32-32). Still, there is one flat layer of 32 units and the classification layer at the end of the network. For example, the description k30 × 30-cnn32-32-nn32-32 states for the model, in which first the Kohonen network of size 30 × 30 is used to calculate the U-images, which are then used as input to CNN with two convolutional layers wit 32 filters each, then after flattening there are two flat layers with 32 units each. The network ends with the usual layer of 32 units and the classification layer. Figure 8 presents such a model. Note that Kohonen network itself is not presented.

Table 4 presents results for convolutional neural networks with inputs as U-images from Kohonen map. Additional hidden flat layers were used.

### 4.5. Multiple Input Neural Network

In this section, we test the idea of using both types of input, i.e., the original feature vectors and the U-images produced by Kohonen network are used to train the classification model. The neural networks in this sections have two branches. The first one is the series of convolutional layers with U-images as input. The second branch is composed of the ordinary flat hidden layers (just one in the case of the tests reported here) and accepts the original feature vectors as input. These two branches, after the necessary flattening operation, are then merged and processed together by a number of flat layers. For each Kohonen network size (k16 × 16, k20 × 20, k24 × 24 and k30 × 30), we tested the following multiple input CNN architectures: 1xconv-1 × 32-1 × 32, 2xconv-1 × 32-1 × 32, 2xconv-1 × 32-2 × 32, 3xconv-1 × 32-2 × 32. The first part of the description gives the number of convolutional layers (one, two or three as 1xconv, 2xconv or 3xconv) in the first branch. They are not separated by max pooling in this case. Each convolutional layer has 32 3 × 3 filters. The output of the last convolutional layer is flatten and processed by one flat layer of 32 ReLU units. This constitutes the output of the first branch. The second branch accepts the original features and processes them with one hidden layer of 32 ReLU units (second part in the description, 1 × 32). This constitutes the output of the second branch. The output of both branches are concatenated and processed by the number of hidden flat layers given in the third part of the description. For example, 2 × 32 means that there are two flat layers, each with 32 ReLU units. Given the bigger size of the network, we add Dropout layer before the classification layer.

Figure 9 presents an example model described as k30 × 30-3xconv-1 × 32-2 × 32 configured for spambase dataset (57 original features). Note, that the Kohonen network itself is not visualized.

Table 5 presents results for multiple input neural networks.

### 4.6. Summary of the Results

Table 6 presents the summary of the results. Presented are mean test errors in %. Best results are underlined.

In order to statistically evaluate the results, we took the best results for each classification problem from both groups of methods, i.e., ordinary neural networks, and neural networks of any architecture which uses Kohonen-based preprocessing. To test the null hypothesis that there is no difference in the results, we apply the two-sided Wilcoxon test. The null hypothesis in this test is that the median of the differences is zero against the alternative that it is different from zero. The *p*-value is 0.0229, so we reject the null hypothesis, concluding that there is a difference in the results between the neural networks which use Kohonen network-based preprocessing and those which do not. To confirm that the median of the differences can be assumed to be non-zero in favour of Kohonen-based models, we use one-sided Wilcoxon test. It gives the *p*-value of 0.0115. Hence, we conclude that Kohonen-based models give statistically better results on the tested classification problems.

Based on the values given in Table 6, we can draw interesting conclusions about the usefulness of the original attributes compared to the proposed U-Image representation. Models using only the original features (column Ordinary NN) were the best ones in four classification problems. On the other hand, models using only the U-image representation as input (columns Kohonen-CNN and Hybrid) obtained the best results in seven cases. This fact proves in favor of the proposed method. Undoubtedly, combining the original attributes and the new U-image representation can be an excellent approach to some classification problems. This worked best in five cases.

We can also count the cases in which the best model utilized the original attributes, regardless of whether it also used the U-image representation (columns Ordinary NN and Multiple input). There are nine problems where the best model used the original features. We can make similar analysis for the U-image representation, i.e., we count the problems in which the best model utilized this representation, regardless of whether it used the original features (columns Kohonen-CNN, Hybrid, and Multiple input). There are twelve such problems. Thus, according to this criterion the presented method is generally worth considering , too.

The presented results show that the proposed method often improves the results compared to the ordinary feed-forward neural network and the use of only original features. It can be advantageous when searching for a better model by checking different architectures of the ordinary neural network does not lead to improvement.

**Figure 10:**
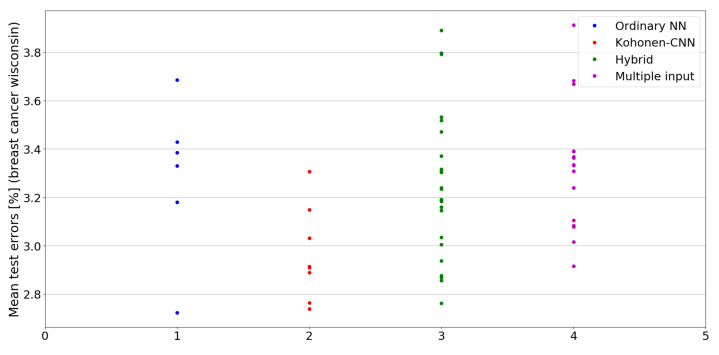
Cross-validation test errors for different architectures for breast cancer wisconsin dataset.

**Figure 11:**
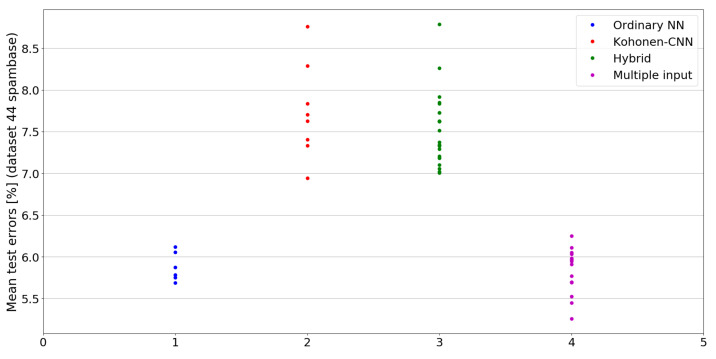
Cross-validation test errors for different architectures for dataset 44 spambase.

**Figure 12:**
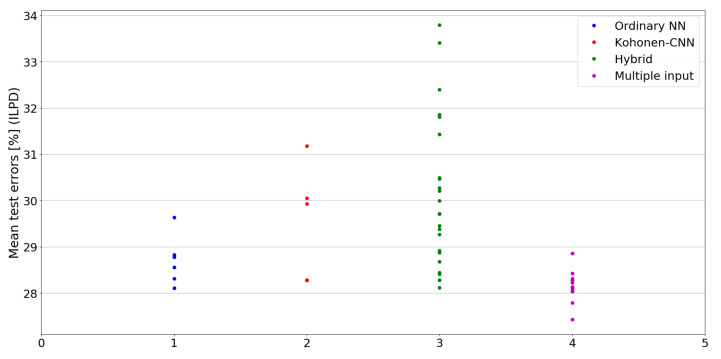
Cross-validation test errors for different architectures for ILPD dataset.

**Figure 13:**
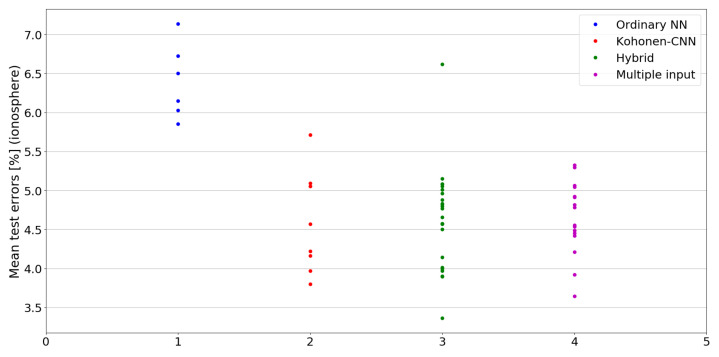
Cross-validation test errors for different architectures for ionosphere dataset.

**Figure 14:**
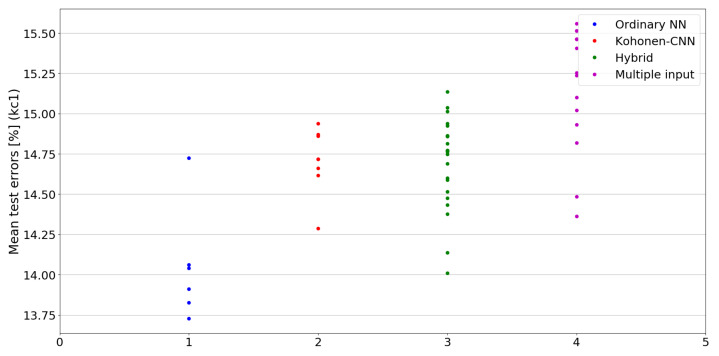
Cross-validation test errors for different architectures for kc1 dataset.

**Figure 15:**
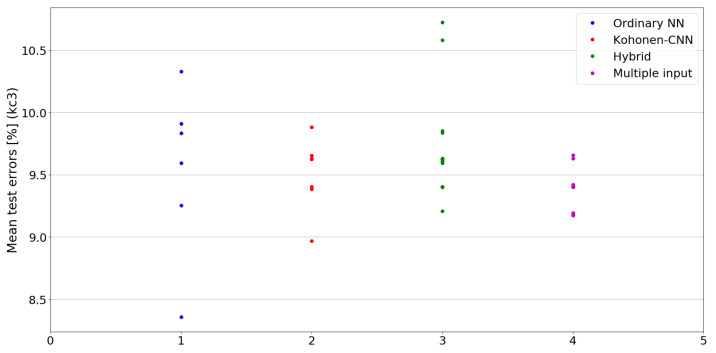
Cross-validation test errors for different architectures for kc3 dataset.

**Figure 16:**
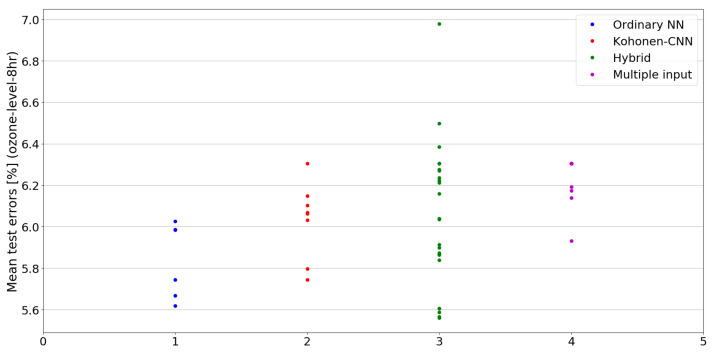
Cross-validation test errors for different architectures for ozone-level-8hr dataset.

**Figure 17:**
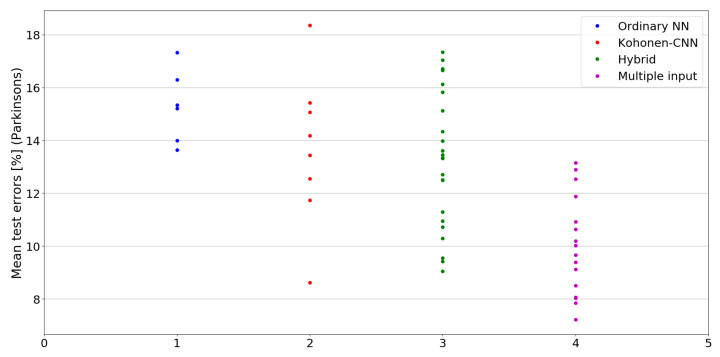
Cross-validation test errors for different architectures for Parkinsons dataset.

**Figure 18:**
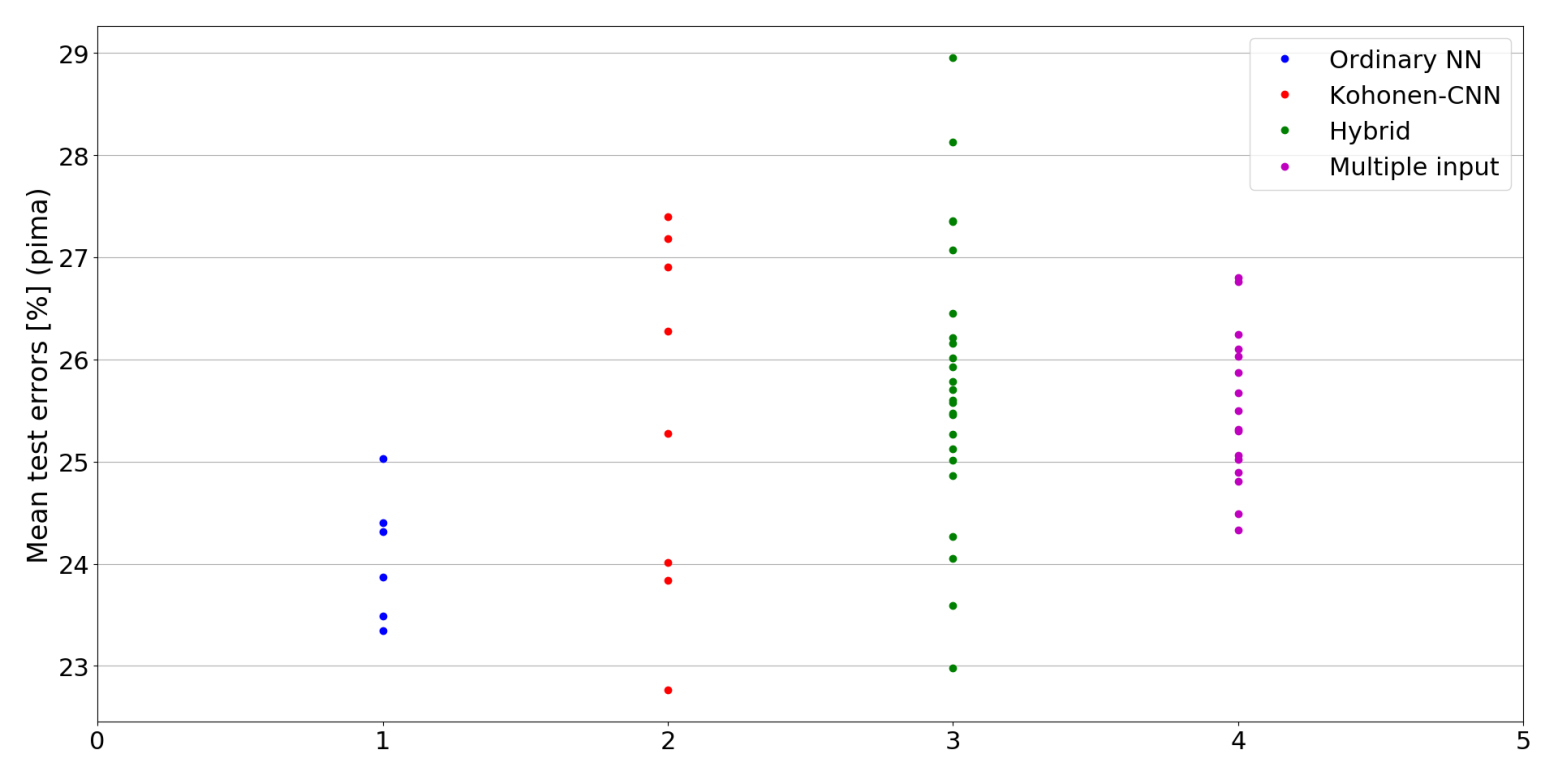
Cross-validation test errors for different architectures for pima dataset.

**Figure 19:**
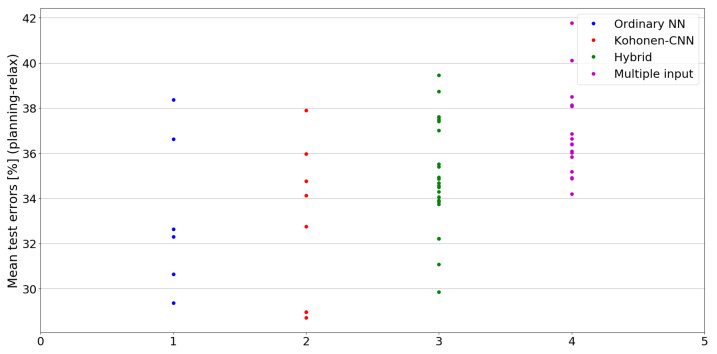
Cross-validation test errors for different architectures for planning-relax dataset.

**Figure 20:**
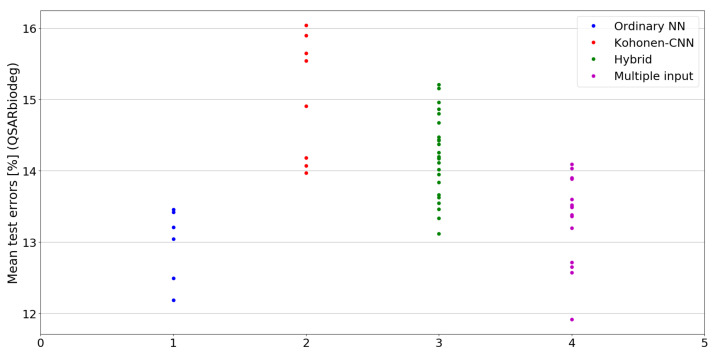
Cross-validation test errors for different architectures for QSARbiodeg dataset.

**Figure 21:**
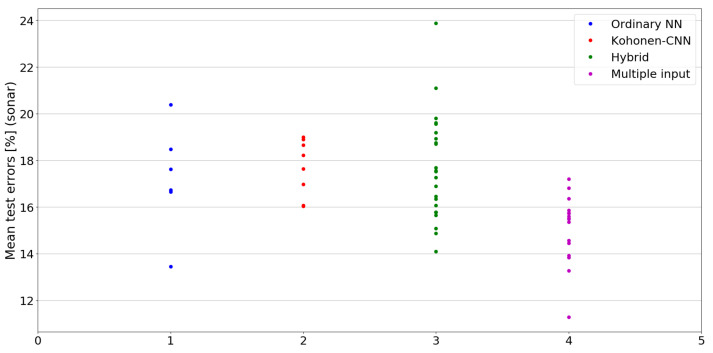
Cross-validation test errors for different architectures for sonar dataset.

**Figure 22:**
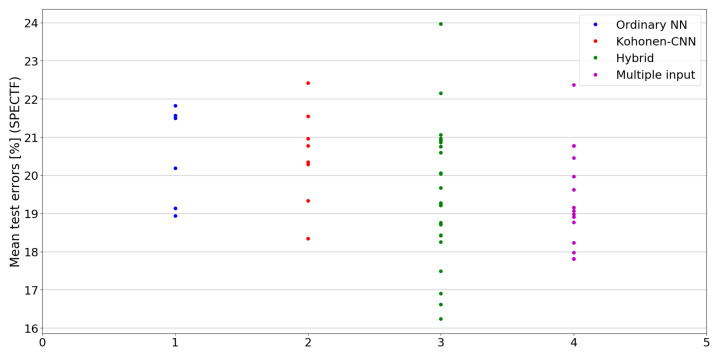
Cross-validation test errors for different architectures for SPECTF dataset.

**Figure 23:**
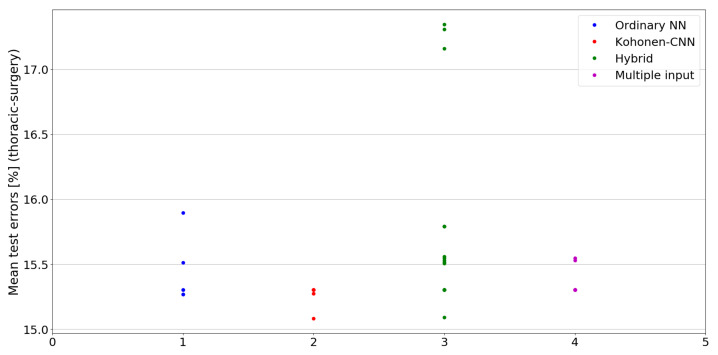
Cross-validation test errors for different architectures for thoracic-surgery dataset.

**Figure 24:**
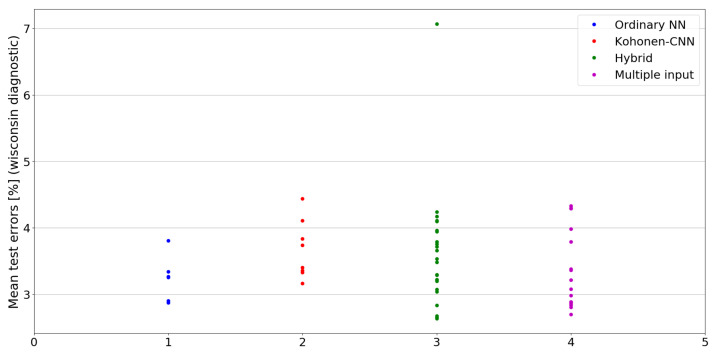
Cross-validation test errors for different architectures for wisconsin diagnostic dataset.

**Figure 25:**
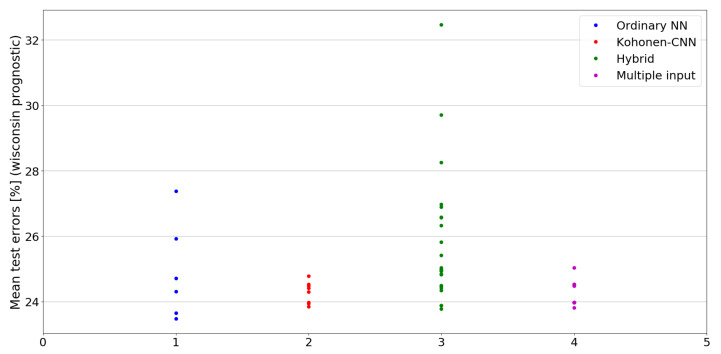
Cross-validation test errors for different architectures for wisconsin prognostic dataset.

The benefit of the proposed method is sometimes higher and sometimes lower.It is natural to ask how difficult it is to find the correct neural network architecture. A general answer to this question is impossible, however Figure 10, Figure 11, Figure 12, Figure 13, Figure 14, Figure 15, Figure 16, Figure 17, Figure 18, Figure 19, Figure 20, Figure 21, Figure 22, Figure 23, Figure 24 and Figure 25 show mean cross-validation test errors for each neural architecture tested in each group. There were 6, 8, 24 and 16 different configurations of ordinary feed-forward neural networks, Kohonen-CNN, hybrid (with additional hidden flat layers) and multiple input, respectively. The details are presented in previous sections. We can observe that the degree of the usefulness of the proposed technique varies among the classification problems. For the Parkinsons problem the proposed method brought about the most remarkable improvement compared to classic networks. In Figure 17, we see that the combination of the original attributes with the U-image representation worked out especially well in this case. Some U-image-only networks (Kohonen-CNN and Hybrid) performed better but some performed worse. Nevertheless, the potential of the proposed method is visible here. Figure 22 shows a similar plot for the SPECTF problem, in which the proposed method also gave better results, but the improvement was not as significant as for the Parkinsons problem. The proposed U-image representation works very well for ionosphere, too (Figure 13). On the other hand, for dataset 44 spambase (Figure 11) the need of the original features seems to be obvious. In general, finding the right U-image-based architecture is not straightforward, but the improvement is possible in many cases. The proposed method allows searching for additional configurations compared to using only the feed-forward networks.

### 4.7. Preliminary Results for Particle Data and Directions of Future Work

In addition to the numerical experiments presented in the previous section, we conducted additional preliminary attempts to develop a classifier for another complex problem presented in [19]. This classification problem concerns collisions at high-energy particle colliders. It is defined by the need to solve difficult signal-versus-background classification. There are 21 features which are kinematic properties measured by the particle detectors in the accelerator and seven features (derived by physicists) which are functions of the first 21 features. Features in this problem are an example of sensory measurements, which cannot be directly applied to convolutional layers. These data are available in the UCI repository. The total number of samples available in this collection is 11,000,000.

Given the large size of the data, we only conducted preliminary numerical experiments. We only considered a subset of the available data, i.e., the first 100,000 examples as training data. We used all 500,000 test examples as recommended in the original study. Cross-validation was not used. As in the original study, the AUC value was the metric we used to compare the models. The Kohonen network sizes used were 30 × 30, 40 × 40, and 50 × 50. Only a multiple-input network architecture was used. By checking several architectures, the best results were obtained for a network with three convolutional layers with 32 filters in the first branch and one hidden layer with 32 ReLU neurons in the second branch. The mean AUC of the ten runs was 0.7221, with the highest of 0.7278. Compared to these results, a regular three-layer neural network with 32 ReLU neurons in each layer gave an average AUC value of 0.7211 and the highest 0.7264 (several architectures were tested, here we report the best results). Thus, we see that the results have improved after applying the Kohonen network and convolutional layers. The improvement, however, is slight and points to the need for further improvements to the method. The results are also worse than in the original publication, which may be due to the use of only a subset of the available data. Therefore, further work will adapt the algorithm to handle large data sets and parallelize the calculations.

It is also worth noting that only one Kohonen network of a given size was always used in the solution presented in this paper. This means that the input of the first convolutional layer is always an image with one channel of size equal to the size of the Kohonen network. The next stage in developing the presented method will be the use of many Kohonen networks, each of which will provide one mapping, i.e., one image channel constituting the input for the convolution layer. This is justified, as after starting from random weight values, each Kohonen network training process ends with a different mapping of multidimensional data to 2D space. The variety of individual channels can also be obtained by changing the parameters of the Kohonen network training algorithm, neighborhood definition, or even other training algorithms not presented in this paper. Various mappings can help present non-sequential but multidimensional and complex data as images with different channels. It will further facilitate the convolutional network discovering complex relationships in the data.

It is also worth mentioning the phenomenon of dead neurons in the Kohonen network and the resulting restriction on the size of the network. A dead neuron is one that is not a BMU for any training example. Such a phenomenon will undoubtedly occur if the number of training examples is smaller than the number of neurons in the Kohonen network. For example, in a 30 × 30 Kohonen network, we have 900 neurons. In this case, theoretically, the minimum number of training examples should be at least also equal to 900, but in practice, it should be much larger. It follows that only for large training sets, the proposed method can provide input images with higher resolutions.

## 5. Conclusions

This work presents a new way of using the computational capabilities of convolutional neural networks. These networks have been used many times with success in problems in which data are sequential (time series) or are strongly spatially correlated (images, movies). For data that do not have these properties, the use of the CNN network is not justified. In this work, it was proposed to use the Kohonen network and the newly proposed U-image object in order to transform the feature vectors into an image accepted as the input signal of the convolution layer. On the example of selected machine learning problems, it was shown that the proposed method leads to better results. The possibilities for further development of the proposed approach were also indicated. Considering how successful the application of the CNN in image processing problems was, the proposed method may prove to be an interesting alternative when building classification models for non-sequential data.

## Figures and Tables

**Table 1:** Machine learning test databases used in experiments.

No	Database	Examples	Features	Majority Class Ratio
1	breast cancer wisconsin	683	9	0.6501
2	dataset 44 spambase	4601	57	0.6060
3	ILPD	579	10	0.7150
4	ionosphere	351	33	0.6410
5	kc1	2109	21	0.8454
6	kc3	458	39	0.9061
7	ozone-level-8hr	2534	72	0.9369
8	parkinsons	195	22	0.7538
9	pima	768	8	0.6510
10	planning-relax	182	12	0.7143
11	QSARbiodeg	1055	41	0.6626
12	sonar	208	60	0.5337
13	SPECTF	267	44	0.7940
14	thoracic-surgery	470	16	0.8511
15	wisconsin diagnostic	569	30	0.6274
16	wisconsin prognostic	194	32	0.7629

**Table 2:** Results for ordinary feed-forward neural networks.

No	Database	Mean Test Error [%]	StD [%]	Architecture
1	breast cancer wisconsin	2.72	1.88	16-32
2	dataset 44 spambase	5.69	1.35	32
3	ILPD	28.11	9.26	32-32
4	ionosphere	5.85	3.54	16-32
5	kc1	13.73	2.08	32-16-32
6	kc3	8.36	3.90	16-16-32
7	ozone-level-8hr	5.62	1.45	16-32
8	parkinsons	13.63	6.82	32-32-32
9	pima	23.34	4.56	32
10	planning-relax	29.37	8.84	32
11	QSARbiodeg	12.19	2.62	16-16-32
12	sonar	13.45	6.02	32-16-32
13	SPECTF	18.93	10.31	32-32-32
14	thoracic-surgery	15.27	5.67	32-32
15	wisconsin diagnostic	2.87	1.44	32-32-32
16	wisconsin prognostic	23.48	13.12	32

**Table 3:** Results for convolutional neural networks with inputs as U-images from Kohonen map.

No	Database	Mean Test Error [%]	StD [%]	Architecture
1	breast cancer wisconsin	2.74	1.88	k20 × 20-cnn16
2	dataset 44 spambase	6.94	1.06	k30 × 30-cnn32-16
3	ILPD	28.28	7.05	k16 × 16-cnn16
4	ionosphere	3.80	3.08	k30 × 30-cnn32-32
5	kc1	14.29	1.96	k20 × 20-cnn32-16
6	kc3	8.97	4.46	k20 × 20-cnn32-16
7	ozone-level-8hr	5.74	1.51	k16 × 16-cnn32-16
8	parkinsons	8.61	3.70	k30 × 30-cnn32-32
9	pima	22.76	4.60	k16 × 16-cnn16
10	planning-relax	28.71	9.73	k16 × 16-cnn16
11	QSARbiodeg	13.97	3.82	k16 × 16-cnn32-16
12	sonar	16.03	5.47	k30 × 30-cnn32-16
13	SPECTF	18.35	7.64	k30 × 30-cnn32-16
14	thoracic-surgery	15.08	6.37	k30 × 30-cnn32-32
15	wisconsin diagnostic	3.16	1.90	k30 × 30-cnn32-32
16	wisconsin prognostic	23.85	12.73	k24 × 24-cnn16

**Table 4:** Results for convolutional neural networks with inputs as U-images from Kohonen map. Additional hidden flat layers were used.

No	Database	Mean Test Error [%]	StD [%]	Architecture
1	breast cancer wisconsin	2.76	2.10	k16 × 16-cnn16-nn32
2	dataset 44 spambase	7.00	0.62	k20 × 20-cnn32-32-nn32
3	ILPD	28.12	6.96	k16 × 16-cnn32-32-nn32-32
4	ionosphere	3.36	2.55	k24 × 24-cnn32-32-nn32
5	kc1	14.01	2.24	k30 × 30-cnn16-nn32
6	kc3	9.21	4.73	k16 × 16-cnn16-nn32-32
7	ozone-level-8hr	5.56	1.30	k16 × 16-cnn32-nn32
8	parkinsons	9.05	3.08	k30 × 30-cnn32-32-nn32
9	pima	22.98	4.26	k16 × 16-cnn16-nn32
10	planning-relax	29.85	9.51	k16 × 16-cnn16-nn32
11	QSARbiodeg	13.12	2.49	k30 × 30-cnn32-32-nn32-32
12	sonar	14.10	7.29	k16 × 16-cnn32-32-nn32-32
13	SPECTF	16.24	8.47	k20 × 20-cnn16-nn32
14	thoracic-surgery	15.09	6.22	k24 × 24-cnn32-32-nn32
15	wisconsin diagnostic	2.63	1.54	k24 × 24-cnn16-nn32
16	wisconsin prognostic	23.78	11.15	k30 × 30-cnn32-nn32-32

**Table 5:** Results for multiple input neural networks.

No	Database	Mean Test Error [%]	StD [%]	Architecture
1	breast cancer wisconsin	2.92	1.93	k24 × 24-1xconv-1 × 32-1 × 32
2	dataset 44 spambase	5.26	1.16	k16 × 16-2xconv-1 × 32-1 × 32
3	ILPD	27.43	6.95	k20 × 20-3xconv-1 × 32-2 × 32
4	ionosphere	3.64	3.45	k30 × 30-2xconv-1 × 32-1 × 32
5	kc1	14.36	1.91	k16 × 16-1xconv-1 × 32-1 × 32
6	kc3	9.18	4.67	k16 × 16-1xconv-1 × 32-1 × 32
7	ozone-level-8hr	5.93	1.03	k16 × 16-2xconv-1 × 32-2 × 32
8	parkinsons	7.21	4.37	k30 × 30-2xconv-1 × 32-2 × 32
9	pima	24.33	5.36	k20 × 20-2xconv-1 × 32-2 × 32
10	planning-relax	34.20	6.26	k24 × 24-2xconv-1 × 32-1 × 32
11	QSARbiodeg	11.91	2.71	k16 × 16-1xconv-1 × 32-1 × 32
12	sonar	11.28	7.22	k24 × 24-3xconv-1 × 32-2 × 32
13	SPECTF	17.81	5.03	k16 × 16-2xconv-1 × 32-2 × 32
14	thoracic-surgery	15.30	6.10	k16 × 16-1xconv-1 × 32-1 × 32
15	wisconsin diagnostic	2.70	2.17	k16 × 16-2xconv-1 × 32-1 × 32
16	wisconsin prognostic	23.81	12.28	k24 × 24-1xconv-1 × 32-1 × 32

**Table 6:** Summary of the results. Presented are mean test errors in %. Best results are underlined.

No	Database	Ordinary NN	Kohonen-CNN	Hybrid	Multiple input
1	breast cancer wisconsin	2.72	2.74	2.76	2.92
2	dataset 44 spambase	5.69	6.94	7.00	5.26
3	ILPD	28.11	28.28	28.12	27.43
4	ionosphere	5.85	3.80	3.36	3.64
5	kc1	13.73	14.29	14.01	14.36
6	kc3	8.36	8.97	9.21	9.18
7	ozone-level-8hr	5.62	5.74	5.56	5.93
8	parkinsons	13.63	8.61	9.05	7.21
9	pima	23.34	22.76	22.98	24.33
10	planning-relax	29.37	28.71	29.85	34.20
11	QSARbiodeg	12.19	13.97	13.12	11.91
12	sonar	13.45	16.03	14.10	11.28
13	SPECTF	18.93	18.35	16.24	17.81
14	thoracic-surgery	15.27	15.08	15.09	15.30
15	wisconsin diagnostic	2.87	3.16	2.63	2.70
16	wisconsin prognostic	23.48	23.85	23.78	23.81

## Data Availability

https://archive.ics.uci.edu/ (accessed on 1 September 2021).
